# Chitinase 3-Like 1 Protein Levels Are Elevated in *Schistosoma haematobium* Infected Children

**DOI:** 10.1371/journal.pntd.0001898

**Published:** 2012-11-08

**Authors:** Laura J. Appleby, Norman Nausch, Claire D. Bourke, Nadine Rujeni, Nicholas Midzi, Takafira Mduluza, Judith E. Allen, Francisca Mutapi

**Affiliations:** 1 Institute for Immunology & Infection Research, Centre for Immunity, Infection & Evolution, School of Biological Sciences, University of Edinburgh, Edinburgh, United Kingdom; 2 National Institutes of Health Research, Causeway, Harare, Zimbabwe; 3 Department of Biochemistry, University of Zimbabwe, Mount Pleasant, Harare, Zimbabwe; Uniformed Services University, United States of America

## Abstract

**Background:**

Currently there are few studies characterising the nature and aetiology of human schistosome-related inflammatory processes. The aim of this study was to determine the relationship between Chitinase 3-like 1 (CHI3L1), also known as YKL-40, a molecule associated with inflammatory processes, and schistosome infection, morbidity and systemic cytokine levels.

**Methods:**

Serological levels of CHI3L1 and a panel of cytokines (IFN-y, IL-4/5/6/9/10/13 and 17) were measured in two Zimbabwean populations resident in a high and low schistosome infection area. CHI3L1 levels were related to schistosome infection, haematuria status and cytokine levels after allowing for confounding variables. The effect of antihelminthic treatment with praziquantel on CHI3L1 levels was determined in 246 participants 6 weeks post-treatment.

**Results:**

CHI3L1 levels increased with age in both areas but were significantly higher in the high infection areas compared to the low infection area. CHI3L1 levels were also higher in infected compared to uninfected individuals with this difference being significant in the youngest age group. Curative antihelminthic treatment resulted in a significant decrease in CHI3L1 levels. Of the cytokines, only IL-10 and IL-17 had a significant association with CHI3L1 levels, and this association was negative.

**Conclusions:**

Serum CHI3L1 levels differ between infected and uninfected people before and after antihelminthic treatment. The greatest difference occurs in the youngest age group, in keeping with the period when schistosome-related pathological processes are initiated. Following from previous studies in non-infectious diseases showing that CHI3L1 is a biomarker for the inflammatory process, this study suggests that the potential for CHI3L1 as a biomarker for schistosome-related pathology should be explored further.

## Author Summary

Over 100 million people, mainly living in sub-Saharan Africa, are infected with the blood fluke *Schistosoma haematobium*. Morbidity includes anaemia, malnutrition, impaired cognition and physical growth as well as pathology of the urogenital tract. Urogenital morbidity is initiated by eggs laid at the site of infection causing mechanical damage and leading to haematuria, or blood in the urine. Eggs also secrete antigens, triggering a protective granuloma to form around the eggs which can lead to fibrosis and organ failure. Currently there are few non invasive markers of schistosome-related pathology hampering diagnosis and evaluation of control programmes. Chitinase 3-like 1 (CHI3L1) has been associated with several inflammatory disorders involving tissue remodelling. In this study we assessed the levels of CHI3L1 in a group of individuals from a schistosomiasis endemic area in Zimbabwe. We showed that CHI3L1 is elevated in schistosomiasis infected individuals over uninfected individuals, with the strongest association seen in the youngest age group who have a shorter history of schistosome infection. Treating individuals with a drug that eliminates the adult worm leads to a decrease in CHI3L1 in infected individuals. These results suggest that the potential for CHI3L1 as a biomarker for schistosomerelated pathology should be explored further in different human populations.

## Introduction

Urogenital schistosomiasis, caused by the blood fluke *Schistosoma haematobium*, is one of the world's most prevalent human parasitic diseases. Morbidity includes anaemia, malnutrition, impaired memory cognition and physical growth [1], and immune-mediated pathology in the urogenital tract, the kidneys and ureters [2]. Immunopathology commences with the laying of eggs in the blood vessels at the site of infection where they cause damage to the bladder walls and genital tissues in the form of lesions in blood vessels and tissues leading to the common symptom of haematuria. Antigens secreted by these eggs induce a Th2 response, involving secretion of interleukin-4 (IL-4), IL-5 and IL-13 [3], [4] and the eventual development of granulomas [5], [6]. Resolution of the granuloma involves deposition of collagen and extra-cellular matrix components, which are the source of fibrosis [5], [7], [8]. In young children infected with *S. haematobium* egg deposition is associated with more acute inflammatory symptoms, visible haematuria, anaemia and a high egg output in what is known as the active stage of disease [7]. This progresses with age to an inactive stage characterised by a drop in urine egg counts as fewer eggs are excreted but more become trapped and calcified in tissues. It is during the inactive stage of disease that signs of extensive and sometimes irreversible fibrotic pathology can be detected [7], while in those still acquiring active infection most of the schistosomiasis associated morbidity is reversible upon antihelminthic treatment [9]. Schistosome control programmes aim to reduce morbidity by reducing infection intensity and their success is monitored through egg counts [10], [11]. However, egg counts as a measure of disease burden can be misleading as the relationship between morbidity and infection intensity can be non linear, for example there can be a discrepancy between an individual with a high worm burden displaying less pathology than an individual with a low worm burden [10], [12]. Haematuria, has been used in numerous studies as both a marker for infection and onset of pathology [10], it is particularly effective in detecting infection in younger age groups. However, haematuria can have a low sensitivity rate [13]. Ultrasonography provides a more reliable method for assessing pathology in schistosomiasis infection [10] but operational logistics, such as the requirement for trained personnel and a consistent power source, make it an unsuitable tool for large scale or rural field studies [14]. In addition, despite its effectiveness in detecting late stage disease, ultrasound can fail to detect the earlier pathological changes associated with infection mediated inflammation [15]. Identification of biomarkers that can detect early pathological changes related to schistosomiasis infection, and changes associated with treatment, would provide an invaluable tool in monitoring schistosome control programmes [11].

Chitinase-like proteins are a characteristic feature of multiple helminth infection models [16], [17]. Chitinase 3-like 1 (CHI3L1), also known as human cartilage glycoprotein 39 (HCgp-39), and YKL-40, is a human chitinase-like chitin-binding lectin with no chitinase enzymatic activity [16], [17]. It is expressed by numerous cell types and has been associated with collagen and extra-cellular matrix formation [18] as well as with a wide range of diseases characterised by inflammation and tissue remodelling including asthma, arthritis, numerous cancers and liver fibrosis [19], [20], [21]. Recently CHI3L1 has also been linked to schistosome related hepatic fibrosis with *S. japonicum* infection [22]. In order to evaluate the potential of CHI3L1 as a pathology marker in urogenital schistosomiasis, and to investigate the nature of pathological changes associated with infection, the association of serum CHI3L1 levels with schistosome infection status, prevalence and history of exposure to infection was determined in individuals from three villages in rural Zimbabwe with differing levels of *S. haematobium* endemicity. Comparing villages of different endemicity allows a comparative approach separating the effects of history of schistosome infection from current infection levels, both factors that can potentially influence CHI3L1 levels [23]. The effect of curative antihelminthic treatment on CHI3L1 was determined in a post-treatment follow-up focussed on the village with highest endemicity. In addition we assessed the protein's relationship with a panel of systemic cytokines encompassing the range of Th1, Th2, Th17 and T-regulatory responses associated with schistosome infection and pathology (see [24], [25] for review), thus allowing CHI3L1 levels to be related to the immune environment in the host relative to their immune phenotype.

## Materials and Methods

### Ethical Statement

The study was conducted in the Mashonaland East Province of Zimbabwe where *S. haematobium* is endemic. Ethical approval was received from the University of Zimbabwe's Ethics Review Board (UZERB) and the Medical Research Council of Zimbabwe (MRCZ), and permission to conduct the study was obtained from the Provincial Medical Director. The study design, aims and procedures were explained in the local language, Shona, prior to enrolment. Written consent was obtained from participants or their guardians prior to taking part in the study.

### Study area and population

The study was conducted within schools, recruiting school children and community members from three villages and details are described elsewhere [26]. Of the three villages Magaya (17°37′21″S 31°54′36″E) and Chipinda (17°41′46″S 31°56′03″E) are considered high infection areas (HIA) for schistosome infection, while Chitate (17°39′45″S 31°53′21″E) is a low infection area (LIA) as defined by the World Health Organization (WHO) [11]. The area has a low prevalence of soil transmitted helminths [27], and the residents are subsistence farmers with frequent contact with infected water for purposes of bathing, washing and collecting water. *Plasmodium falciparum* is the predominant species of malaria in Zimbabwe, and in this region malaria transmission is largely unstable in nature, classified as low and sporadic [28], [29], giving a low prevalence of co-infection between schistosome infection and *Plasmodium* infection in the study population.

### Sample collection

From each participant a stool and urine specimen was collected on three consecutive days and examined microscopically. Urine samples were examined for *S. haematobium* infection and stool samples were examined for *S. mansoni* and soil transmitted helminths using standard techniques [30], [31]. Urine samples from children were examined for visible haematuria and designated positive when haematuria was observed or negative when no haematuria was observed. Macrohaematuria was confirmed on a different urine sample by dipstick (Uripath, Plasmatec, UK). 5 ml venous blood was collected and processed using routine methods to collect serum which was frozen at −20°C prior to freighting to Edinburgh for serological assays. Following parasitology sample collection, participants were offered treatment with the antihelminthic drug praziquantel at the recommended dose of 40 mg/kg of body weight and treatment efficacy was checked 6 weeks later during follow-up parasitology and serological surveys. Treatment of the three communities was staggered by 6 weeks for the purpose of an untreated control group except in the case of heavily infected people as defined by the WHO [11].

### Inclusion/Exclusion Criteria

In order to be included in this study participants had to meet the following criteria: 1) be lifelong residents of the study area to allow age to be used as a proxy for history of exposure to schistosome infection, 2) have provided at least two urine and two stool samples on consecutive days for parasite detection, 3) have provided a blood sample for serological assays, 4) not have previously received antihelminthic treatment, 5) be negative for co-infections (malaria, intestinal helminths, *S. mansoni* and HIV). From an initial number of 2000 individuals, a total of 859 individuals 2–86 years old, 642 from the HIA and 217 from the LIA, met these criteria. The post-treatment study focused on the HIA where there was a higher prevalence of infection. To be included in the post-treatment study individuals had to meet the criteria above and be confirmed egg negative 6 weeks post-treatment or remain untreated for the control group. 246 participants (110 treated and 136 untreated) fulfilled these criteria and formed the follow-up cohort.

### Serological assays

Serum levels of CHI3L1 were quantified using an ELISA kit from R&D Systems (Cat DY2599, Minneapolis, USA), following the manufacturer's instructions. Systemic levels of IL-10, IL-17, IFN-γ, IL-13, IL-4, IL-5 and IL-6 were measured by ELISA as previously described [32], [33]. All serological assays were performed in duplicate with a positive control on each plate.

### Statistical analysis

Statistical analyses were conducted using the statistical package SPSS version 14.0. To describe the age profiles of schistosome infection and CHI3L1 protein, an analysis of variance (ANOVA) was performed using infection intensity (eggs/10 ml urine) and CHI3L1 levels (ng/ml) log transformed (log_10_(x+1)). The independent variables were age categorised into 6 groups as shown in Table 1. Following these analyses, age was re-categorised to reflect the changing dynamics of rising (2–10 years), peaking (11–20 years) and declining (21+years) infection. Using these age groups the effect of schistosome infection (infection intensity (log transformed (log_10_(x+1)) or infection status (infected vs. uninfected)) and age group on CHI3L1 protein was determined by ANOVA after allowing for the effects of sex and village of residence through sequential sums of squares. The interaction between infection and age groups was also tested. To determine if CHI3L1 levels were higher in children with visible haematuria, the positive cases were matched with age, sex and infection intensity matched controls and the data analysed by t-test.

**Table 1:** Description of study population.

	High Infection Area			Low Infection Area		
Age group (years)	Infection Range (N)	M (N)	F (N)	Infection Range (N)	M (N)	F (N)
2–10	0–865 (273)	136	137	0–28 (74)	37	37
11–15	0–743 (202)	97	105	0–165 (59)	29	30
16–20	0–720 (43)	20	23	0–76 (18)	6	12
21–30	0–52 (21)	1	20	0 (23)	3	20
31–40	0–21 (23)	2	21	0 (14)	14	0
41+	0–14 (80)	9	71	0–8 (29)	5	24
Total	0–865 (642)	265	377	0–165 (217)	80	137

Note. Infection Range (eggs/10 ml urine) and sample sizes, according to age group. Abbreviations: N, sample size; M, male; F, female.

The effect of antihelminthic treatment on CHI3L1 levels was analysed using a repeated measures ANOVA followed by posthoc analysis within each age group. In order to relate systemic cytokine to CHI3L1 levels the number of cytokine variables was reduced using factor analysis. Individual cytokine responses (square-root transformed) with no rotation were used to extract uncorrelated principal components (PC) accounting for a significant proportion of variance in the data (eigenvalues greater than 1) and with a factor loading of >0.5 or <0.5 for one or more of the original cytokine variables. The PCs arising from this procedure were then used in a partial correlation (controlling for age group, sex and residential area) relating the PCs to CHI3L1 levels with the 2-tailed p value from the correlation being reported. P values in all statistical tests were considered significant if p≤0.05.

## Results

### Levels of schistosome infection and CHI3L1 vary with residential area and age

**Figure 1:**
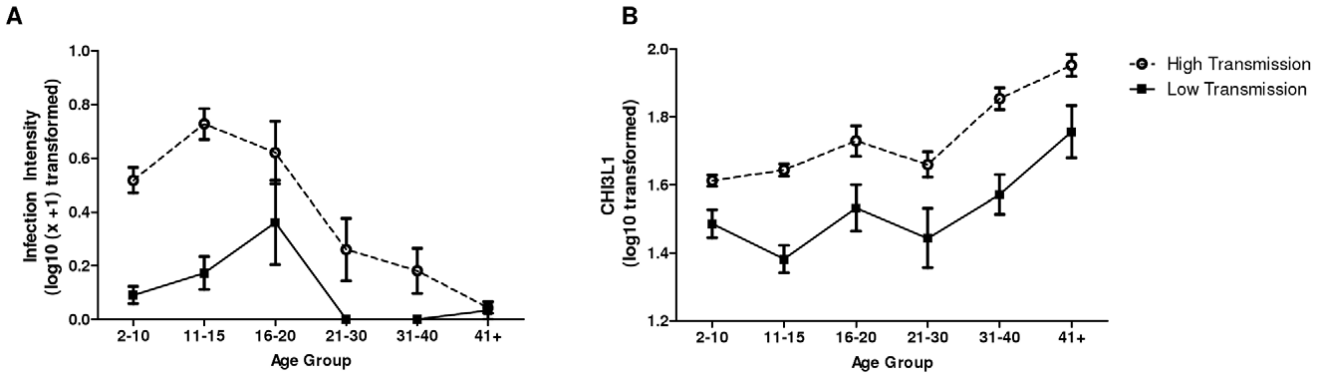
Age profiles for infection intensity and CHI3L1 serum protein levels. Age profiles for the study areas, shown as circles for the high infection area and squares for the low infection areas. Bars represent standard error of the mean. A. Infection intensity exhibiting a peak shift between the two villages. B. Chitinase 3-like 1 protein showing no peak shift between the two villages.

After accounting for sex schistosome infection intensity and prevalence were significantly higher in the HIA (mean infection intensity = 27 eggs/10 ml urine, prevalence = 40%) compared to the LIA (prevalence = 11.1%; mean infection intensity = 2.7 eggs/10 ml urine) (F_1,858_ = 55.158, p<0.001). The schistosome-age profiles differed in the two residential areas resulting in a significant age group*residential area effect (F_5,858_ = 2.446, p = 0.033) giving rise to the ‘peak shift’ [34]; whereby the infection peak occurred at a higher intensity and at an earlier age in the HIA compared to the LIA (as shown in Figure 1A). Overall, after accounting for sex, CHI3L1 levels were also significantly higher in the HIA (mean, 59.9 ng/ml; SEM, 2.03) compared to the LIA (mean, 49.1; SEM 5.15), (F_1,858_ = 59.722, p<0.001). In both villages CHI3L1 levels rose significantly with age group as shown in Figure 1B and Table 2, but no peak shift occurred in CHI3L1 levels.

**Table 2:** F and P values of the factors affecting levels of chitinase 3-like 1 in serum.

Variable	df	F	p value
Sex	1	2.867	0.091
Residential Area	1	69.585**	≤0.000
Age Group	5	25.909**	≤0.000
Infection Status	1	3.552	0.060
Infection Status * Age Group	5	3.017**	0.010
Residential Area * Age Group	5	1.653	0.143
Infection Status * Residential Area	1	1.916	0.167

Note. F and P values from analysis of variance (total degrees of freedom (df) = 858). Sex: Male or Female; Residential areas: high infection area and low infection area; Age groups as described in Table 1; Infection status: egg positive or egg negative.

** Indicates significant p values.

### CHI3L1 levels are elevated in infected individuals, and in haematuric cases

**Figure 2:**
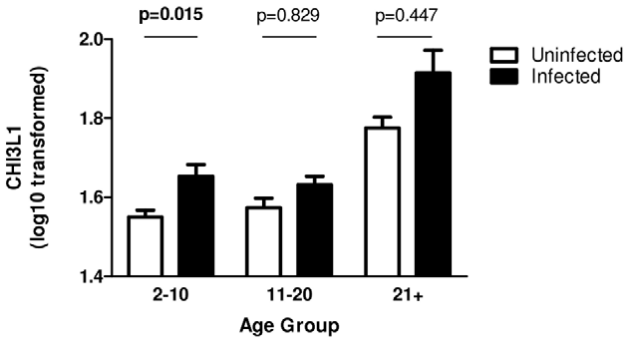
Relationship between CHI3L1 protein and infection status in different age groups. Figure shows mean levels of CHI3L1 protein (log_10_(x+1)). Uninfected individuals (open) compared to infected (solid). Bars represent standard error of the mean. P-values are obtained from posthoc tests after taking into account residential area. Significant p-values are indicated in bold.

**Figure 3:**
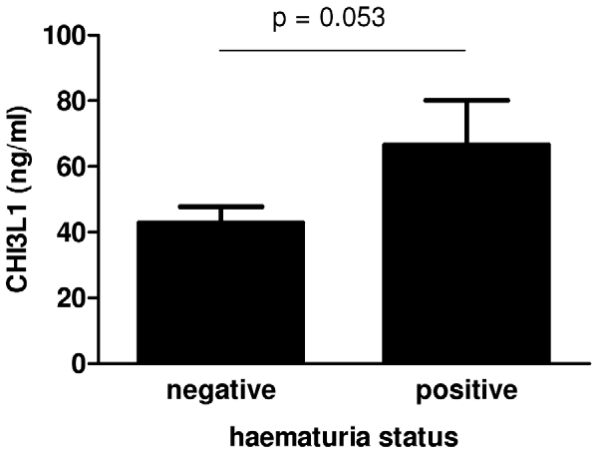
CHI3L1 levels in haematuria positive and haematuria negative individuals. CHI3L1 levels according to haematuria status. P values are obtained from t-test on age, sex and infection status matched individuals.

CHI3L1 levels were higher in infected vs. uninfected people, after allowing for the confounding effects of age and residential area, with this difference being significant in the youngest age group (Table 2, Figure 2). Interestingly, infection intensity, when analysed in the same model, did not significantly affect CHI3L1 levels (F_1,858_ = 2.016, p = 0.156). The pattern was maintained, though was not significant, in the older age groups. Comparison of CHI3L1 levels in 34 children aged 6–16 years showed a higher level of CHI3L1 in the children with visible haematuria compared to the infection matched children who were not presenting with haematuria (t = −1.1662; df = 32; p = 0.053) as shown in Figure 3. Neither the mean age nor the mean infection intensity was significantly different between these 2 groups (t = 0.067; df = 32; p = 0.947 for age, and t = −0.595; df = 32; p = 0.556 for *S. haematobium* infection intensity).

### Praziquantel treatment is associated with a decrease in CHI3L1 level

**Figure 4:**
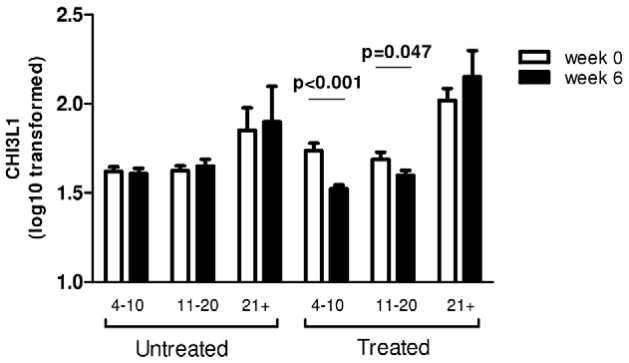
Effect of treatment on CHI3L1 protein levels. Mean CHI3L1 expression within an untreated (n = 136) and a treated population (n = 110) by age group. Week 0 (open), 6 weeks later (solid), bars represent standard error of the mean. Significant p-values from posthoc ANOVA are indicated in bold.

To address the question of whether CHI3L1 is associated with infection, 246 participants from the HIA were followed up for 6 weeks post-treatment. All treated individuals included in this study were negative for infection 6 weeks post-treatment, as determined by egg counts. In treated people, mean CHI3L1 levels declined significantly from 63.7 ng/ml (SEM, 3.06) pre-treatment to 48.2 ng/ml (SEM, 4.75) post-treatment (p<0.001). In contrast, there was no significant change in mean CHI3L1 levels in the untreated group (47.3 ng/ml; SEM = 2.18 at baseline, and 52.3 ng/ml; SEM = 3.52 6 weeks later, p = 0.855). Partitioning the cohort by age group showed that the post-treatment decline was significant in the youngest age groups (2–10 year olds: F_53_ = 23.942, p<0.001; 11–20 year olds F_51,_ = 4.161, p = 0.047), while there was no significant change in the oldest age group (F_6_ = 3.25, p = 0.146) (Figure 4).

### CHI3L1 has a negative relationship with IL-10 and IL-17

The data reduction procedure conducted on the 8 cytokines returned 4 main PCs summarised in Table 3. PC1, accounting for the largest variation in cytokine levels, was composed of Th2-type cytokines (IL-4, IL-5 and IL-9). PC2 was composed of T-regulatory (IL-10) and Th17 (IL-17A) type cytokines. PC3 was composed of IL-13, and PC4 was a pro-inflammatory grouping composed of the Th1 cytokine IFN-γ and the innate cytokine IL-6. Correlating the extracted PCs from the factor analysis with CHI3L1 while controlling for sex, age group, infection area, and infection status revealed a negative association between PC2 and CHI3L1 levels (r = −0.184, p = 0.037). All other PCs showed no significant correlation with CHI3L1 levels PC1 (r = −0.006, p = 0.934), PC3 (r = 0.028, p = 0.755), and PC4 (r = −0.112, p = 0.206).

**Table 3:** Principal component analysis of measured systemic cytokines.

	Principal Component			
	Th2 type (PC1)	Regulatory (PC2)	IL-13 (PC3)	Pro-inflammatory (PC4)
% variation	28.4	20.7	15.6	13.3
IL-4	0.856**	0.131	0.428	−0.088
IL-5	0.849**	0.119	0.458	−0.014
IL-6	−0.081	0.097	0.208	0.842**
IL-9	0.617**	0.304	−0.494	0.158
IL-10	−0.358	0.785**	0.251	0.070
IL-13	0.437	0.303	−0.683**	0.220
IL-17a	−0.329	0.792**	0.123	−0.019
IFN-γ	−0.035	−0.432	0.157	0.517**

Note. Table shows significant principal components (eigenvalue >1) of the data reduction method factor analysis.

** Denotes cytokines with an extracted component of >0.5 or <−0.5.

## Discussion

Infection with schistosome parasites causes a host inflammatory response responsible for much of the disease-associated pathology [3], [4], [5]. As CHI3L1 has been associated with numerous other inflammatory and type-2 diseases we investigated the relationship between CHI3L1 levels and schistosome infection in a population naturally exposed to *S. haematobium* [19], [20], [21].

Our study presents the first comparative analysis of CHI3L1 levels in human populations who are of the same ethnicity and socioeconomic status but differing helminth endemicity. Similar to reports from non-schistosome endemic areas, CHI3L1 showed a significant increase in levels with increasing age [35], a pattern reported to be due to an increasing level of background inflammation associated with ageing and the onset of age-related diseases [35], [36]. In this study we demonstrated that overall levels of CHI3L1 are higher in the HIA compared to the LIA across all age groups. As the populations are similar in ethnicity and in exposure to other common infections such as *Plasmodium*, the only differing characteristic being exposure to schistosome infection [37], this result suggests that the difference in schistosome infection may contribute to differences in CHI3L1 levels and is supported by the observation that schistosome infected people had higher levels of CHI3L1 than uninfected people, independent of residential area. Furthermore CHI3L1 levels were reduced following elimination of adult worms from the body via curative antihelminthic treatment [38], determined via egg count and the disappearance of visible haematuria. Taken together these observations suggest that CHI3L1 levels are related to current levels of infection. The significant interaction between age and infection status with the difference between schistosome-infected and uninfected people being the most apparent in the youngest age group is consistent with a dynamic relationship between infection status and CHI3L1 levels as people age. Other factors confounding the relationship between CHI3L1 and infection status pre- and post- antihelminthic treatment, such as the onset of age-related diseases [35] requires further investigation.

A previous study of schistosome related pathology has suggested that it is infection status rather than infection intensity that is related to pathology [12]. Our results are consistent with this observation in that it was infection status rather than intensity that was associated with CHI3L1 levels. Accordingly, the change in CHI3L1 on clearing infection was not related to pre-treatment infection intensity (data not shown).

To determine if CHI3L1 levels differ in people with visible signs of pathology, we compared levels of the protein in children who had visible haematuria to age and infection intensity matched controls confirmed negative for haematuria. We showed that within infected children, CHI3L1 levels were higher in haematuria positive compared to haematuria negative individuals, suggesting that elevated CHI3L1 levels are associated with infection and morbidity in *S. haematobium* infection. Further studies expanding on the numbers of individuals assessed for haematuria and CHI3L1 levels will be required to confirm this association.

The inflammatory immune response associated with schistosomiasis infection is modulated by adult schistosome worms as well as by the egg antigens [39]. Treatment with PZQ kills the adult schistosome worms and removes their recently laid eggs [40], eliminating the source of inflammation. The decrease in CHI3L1 levels in the treated group may therefore be related to a reduction in inflammatory processes associated with schistosome infection and egg laying, and support previous studies in liver disease that suggests CHI3L1 levels are a bio-indicator of early phases of fibrogenesis [21]. However, while CHI3L1 is associated with inflammation, as yet, its precise function remains unknown. In mice, for instance, inflammatory signals induced by LPS and IFN-γ can both stimulate and inhibit chitinase like protein (CLP) expression in a context dependent manner [41]. As the function of these CLPs is elucidated, it may be possible to make a more educated assessment as to why CHI3L1 is downregulated on clearance of schistosome adult worms.

To determine if elevated CHI3L1 levels are a marker of schistosome-related immunopathology, further studies with larger numbers of people with defined levels of pathology (assessed using current standard markers of schistosome- related immunopathology such as ultrasonography) are necessary. Furthermore, in order to resolve the mechanism behind the observed elevation as well as the relationship between CHI3L1 levels and infection status in the different age groups mechanistic studies on both human and mouse models will be required. One possible explanation is that this could be due to differences in the stage of the pathological processes that the hosts were experiencing, with the younger individuals being at a stage of disease where lesion repair and granuloma formation is actively occurring [7], [9]. Given the lack of significant difference in CHI3L1 levels in infected vs. uninfected individuals in the older age groups, it is unsurprising that treating these individuals does not result in a significant reduction in their CHI3L1 levels. Older individuals have had a lifetime of exposure to infection, and may be experiencing more advanced pathology associated with inactive disease including bladder calcification and a drop in egg excretion [7], [9], as well as a higher background level of CHI3L1 associated with age [35]. There were no macrohaematuria cases observed post-treatment and this is consistent with other studies reporting a reversal/clearance of pathology [42]. These previous studies also reported that the groups experiencing the greatest reduction in morbidity following PZQ treatment are the younger age groups where infection levels are rising but who are not yet suffering from irreversible schistosome pathology [42].

In determining the systemic cytokine environment in the participants relative to CHI3L1, we showed that levels of the measured Th1 and Th2 cytokines were not associated with CHI3L1 levels, perhaps due to the cytokines acting locally at sites of infection [2], but there was an inverse association between CHI3L1 and levels of IL-10 and IL-17A. IL-10 can dampen a damaging pro-inflammatory response [43] and has previously been associated with schistosome infection levels [44] where it can limit pathology during infection as is suggested in the case of bladder pathology [45]. IL-17A is primarily regarded as a pro-inflammatory cytokine and has been found to be associated with numerous inflammatory diseases [46]. Despite being reported to have a role in the schistosome mediated pathological process in murine studies [47], a previous study of people resident in the same region showed less systemic IL-17A levels in *S.* haematobium infected people compared to uninfected individuals [32], and here we describe a negative association with CHI3L1 levels and systemic IL-17A. The relevance and implications of our cytokine findings for CHI3L1 levels in schistosome infection and for schistosome-related pathology remains to be determined in mechanistic studies.

Our study investigated CHI3L1 in a single infection setting; it will be interesting to see if the same relationship with infection status pre- and post- treatment is also observed in *S. mansoni* infection where there is no existing marker for early morbidity [10]. Indeed, in order to fully evaluate CHI3L1 as a potential pathology marker the study must be extended to other settings, including co-infections and differing ethnic populations, and the results compared to existing and candidate pathological markers.

Overall, our study has demonstrated that CHI3L1 levels are associated with schistosome infection status regardless of cumulative history of exposure to infection. Moreover, CHI3L1 is higher in people showing clinical signs of urogenital schistosomiasis (haematuria) compared to infected people presenting no haematuria. Antihelminthic treatment results in a significant reduction of CHI3L1 levels coinciding with the removal of adult schistosome worms. Both before and after curative treatment the difference in CHI3L1 between infected and uninfected people is greatest in the youngest age group where infection levels are rising rapidly, indicating not only a potential mechanism for schistosomiasis associated pathological processes, but also of an early onset of these pathological processes. The associations between CHI3L1 and schistosome infection warrants further investigation to determine the utility of CHI3L1 as a diagnostic marker for schistosome related morbidity as well as a tool for evaluating the impact of *S. haematobium* control programmes.
